# Drug discovery and development scheme for liver-targeting bridged nucleic acid antisense oligonucleotides

**DOI:** 10.1016/j.omtn.2021.10.008

**Published:** 2021-10-19

**Authors:** Fumito Wada, Tsuyoshi Yamamoto, Tadayuki Kobayashi, Keisuke Tachibana, Kosuke Ramon Ito, Mayumi Hamasaki, Yukina Kayaba, Chisato Terada, Asako Yamayoshi, Satoshi Obika, Mariko Harada-Shiba

**Affiliations:** 1 Department of Molecular Innovation in Lipidology, National Cerebral and Cardiovascular Center Research Institute, 6-1 Kishibe-shinmachi, Suita, Osaka 564-8565, Japan; 2 Department of Molecular Pathogenesis, National Cerebral and Cardiovascular Center Research Institute, 6-1 Kishibe-shinmachi, Suita, Osaka 564-8565, Japan; 3 Graduate School of Pharmaceutical Sciences, Osaka University, 1-6 Yamadaoka, Suita, Osaka 565-0871, Japan; 4 Graduate School of Biomedical Sciences, Nagasaki University, 1-14 Bunkyo-machi, Nagasaki 852-8131, Japan

**Keywords:** antisense oligonucleotide, BNA gapmer, AmNA, GalNAc, CEM transfection, PCSK9, LDL-cholesterol, drug delivery, ligand targeting

## Abstract

Antisense oligonucleotides (ASOs) containing bridged nucleic acids (BNAs) have been proven to be very powerful. However, ensuring a reliable discovery and translational development scheme for this class of ASOs with wider therapeutic windows remains a fundamental challenge. We here demonstrate the robustness of our scheme in the context of the selection of ASOs having two different BNA chemistries (2,′4′-BNA/locked nucleic acid [LNA] and amido-bridged nucleic acid [AmNA]) targeting human proprotein convertase subtilisin/kexin type 9 (PCSK9). The scheme features a two-step process, including (1) a unique and sensitive *in vitro* screening approach, called Ca^2+^ enrichment of medium (CEM) transfection, and (2) a ligand-targeted drug delivery approach to better reach target tissues, averting unintended accumulation of ASOs. Using CEM screening, we identified a candidate ASO that shows >70% cholesterol-lowering action in monkeys. An *N*-acetylgalactosamine (GalNAc) ligand then was appended to the candidate ASO to further broaden the therapeutic margin by altering the molecule’s pharmacokinetics. The GalNAc conjugate, HsPCSK9-1811-LNA, was found to be at least ten times more potent in non-human primates (compared with the unconjugated counterpart), with reduced nephrotoxicity in rats. Overall, we successfully showed that our drug development scheme is better suited for selecting clinically relevant BNA-based ASOs, especially for the treatment of liver-associated diseases.

## Graphical abstract

**Figure:**
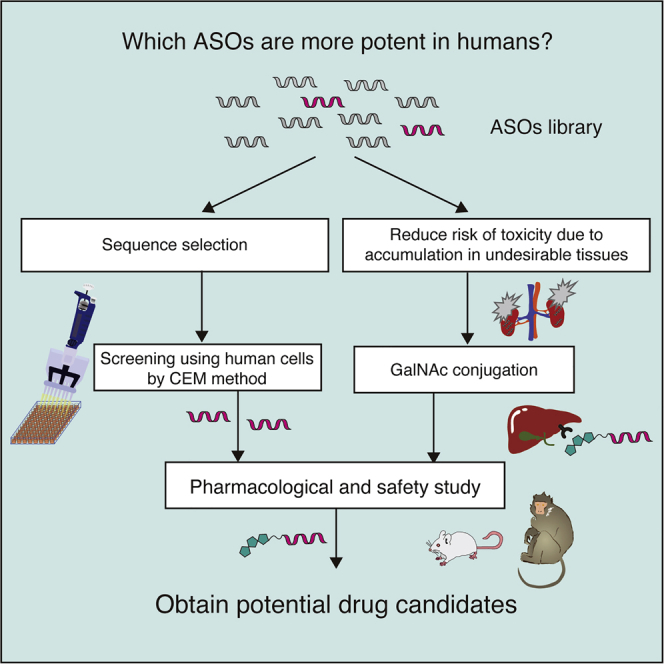

## Teaser

A robust discovery and translational development scheme for selection of clinically relevant BNA-based gapmer ASOs was developed. The scheme features a two-step process, including (1) a unique and sensitive *in vitro* screening approach called CEM and (2) a ligand-targeted drug delivery approach to better reach target tissues, averting unintended side effects.

## Introduction

Nucleic acid drugs such as antisense oligonucleotides (ASOs) and small interfering RNAs (siRNAs) are emerging as pragmatic options for therapeutic modalities, especially for genetic and rare diseases, as the clinical performance of ASOs has been largely improved with innovative chemistry over the past several decades. We have devoted our efforts to developing unique artificial nucleic acids, called bridged nucleic acids or BNAs.^1^ Biochemical analyses have shown that the incorporation of BNAs into ASOs strengthens the binding affinity of such oligonucleotides for their target RNAs. It is now widely accepted that high-affinity, short gapmer ASOs containing these BNA modifications are one particularly promising ASO platform. This high-affinity gapmer configuration has been proven to have enhanced potency, efficiently eliciting RNase H-mediated target RNA cleavage, with alleged propensity to cause higher toxicity (e.g., nephrotoxicity and hepatotoxicity), called “class toxicity.” Thus, generating a reliable discovery/development scheme that is able to properly optimize or select ASO candidates with high potency and a wide therapeutic window by overcoming barriers arising from this class toxicity, as well as species specificity, remains a fundamental challenge for this class of ASOs.

In this context, we previously devised a unique *in vitro* screening methodology that better predicts the *in vivo* efficacy of ASOs; prior to the implementation of this screening approach, we often failed to obtain *in vivo*-active ASOs when using conventional lipofection-based *in vitro* screening.^2^ Our transfection method features the simple enrichment of calcium chloride (to ∼9 mM) in culture medium in the presence of ASOs of interest (with μM to nM activity); we refer to this technique as CEM (Ca^2+^ enrichment of medium). Our previous report validated CEM as an alternative *in vitro* transfection method better suited for selecting *in vivo*-active ASOs.

One reliable approach to avoiding nephrotoxicity is to minimize exposure of the drug to the kidney, given that short oligonucleotides such as high-affinity gapmer ASOs have been shown to preferentially accumulate in the kidney, especially in the proximal tubules.^3^, ^4^, ^5^ We and other groups previously have reported strategies for efficient hepatic delivery of ASOs;^6^, ^7^, ^8^, ^9^, ^10^, ^11^, ^12^, ^13^, ^14^, ^15^, ^16^, ^17^ specifically, the conjugation of a *N*-acetylgalactosamine (GalNAc) ligand has proven to remarkably improve the targeting of ASOs to the liver by reinforcing cellular uptake via receptor-mediated endocytosis while reducing renal accumulation of ASOs. The trimeric form of GalNAc-conjugated ligands is known to serve as an excellent ligand for the asialoglycoprotein receptor that is expressed selectively on hepatocytes.^18^^,^^19^ We previously demonstrated the advantages of our ASOs carrying *trans*-4-hydroxy-l-prolinol-linked GalNAc by showing (in mouse) that this modification reduces the accumulation of ASOs in kidneys while enhancing that in the liver.^11^

Our group has focused on the development of BNA-modified gapmer ASOs for the treatment of dyslipidemia. The proprotein convertase subtilisin/kexin type 9 (PCSK9) is widely accepted as a drug target to reduce the residual risk of cardiovascular disease (CVD).^20^^,^^21^ To date, a small number of clinically relevant therapeutic oligonucleotides targeting *PCSK9* have been described, including ours (Nilsson et al., 2020, Am. Heart Assn., abstract).^22^, ^23^, ^24^ Among the ASOs developed, SPC5001, a 2′,4′-BNA/locked nucleic acid (LNA)-based gapmer ASO against *PCSK9* developed by Santaris Pharma (currently Roche),^23^ had been the most advanced clinical candidate until SPC5001 was shown to induce severe acute kidney injury in healthy volunteers in a Phase I clinical trial,^25^^,^^26^ resulting in the discontinuation of the development of this molecule. The narrow therapeutic window observed for SPC5001 epitomizes the “class effect” of ASOs, posing a challenge to the clinical application of this new class of drugs. There remains great interest in generating ASOs with increased potency and safety.

Our drug discovery scheme has taken the form of a two-step process, including (1) a unique and accurate *in vitro* screening step to obtain more potent *in vivo* activity and (2) a ligand-targeted drug delivery approach to enhance delivery to target tissues, averting unintended accumulation of ASOs, an outcome that often is associated with side effects. Here, we demonstrate our representative discovery/development scheme for high-affinity gapmer ASOs, using *PCSK9* as an exemplary target.

## Results

### *In vitro* screening of ASOs targeting human PCSK9 mRNA

**Figure 1:**
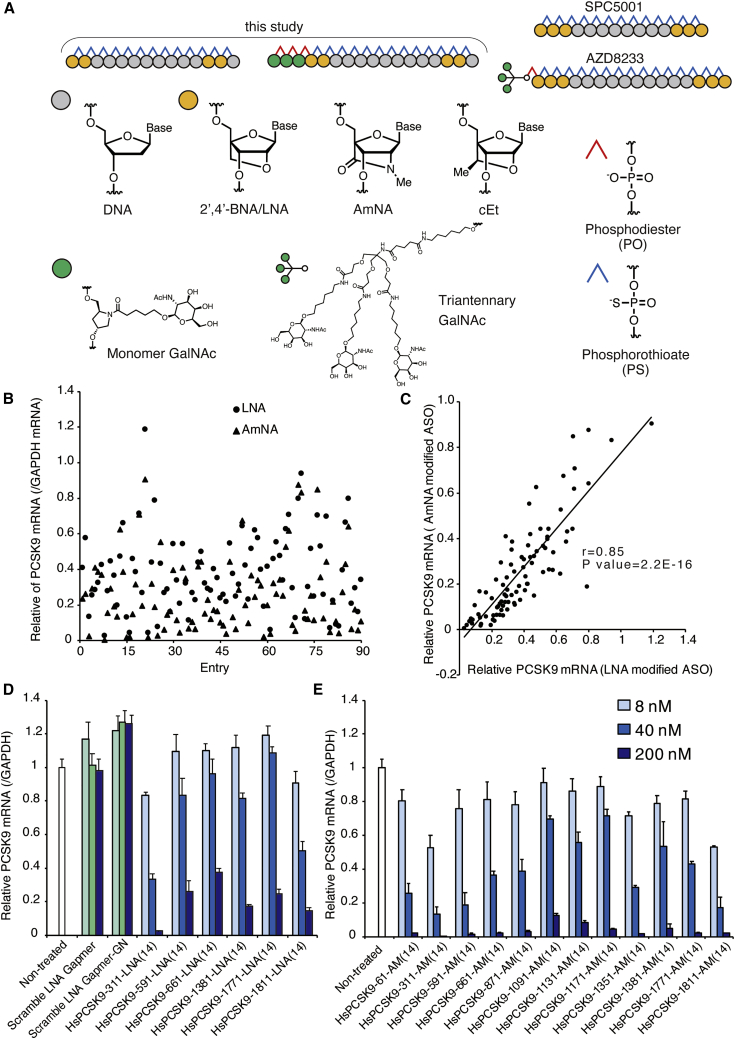
Screening of ASOs targeting human *PCSK9* mRNA using the CEM method in Huh-7 cells (A) Configurations of high-affinity gapmer ASOs and chemical structures of the building blocks. (B) First screen of ASOs. ASOs were transfected at 200 nM with CEM. Human *PCSK9* mRNA knockdown effect of each ASO in Huh-7 cells (n = 1) at 24 h after transfection. (C) To check whether the preferred sequence for each chemical modification is different, the knockdown efficacies of LNA-modified and AmNA-modified ASOs were plotted on the x and y axes, respectively. Pearson’s correlation coefficients (r value) were determined, and single linear regression analysis was performed. (D and E) ASOs identified from the 1^st^ screen were transfected at 8, 40, and 200 nM with CEM (n = 3). Data are presented as mean + SD. For the sequence-scrambled controls in (D), 6.25, 50, and 200 nM were applied. Scramble LNA gapmer: 5ˆ5ˆtˆaˆgˆgˆaˆgˆtˆtˆcˆGˆ5ˆa; scramble LNA gapmer-GN: X_X_X_5ˆ5ˆtˆaˆgˆgˆaˆgˆtˆtˆcˆGˆ5ˆa. For the ASOs, upper-case letters indicate 2′,4′-BNA/LNA and lower-case letters indicate DNA. 5, 5-methyl-cytidine LNA; ˆ, phosphorothioate; _, phosphodiester; X, GalNAc.

To perform an *in vitro* screening using the CEM method, we first designed 180 gapmer-type ASOs using two different BNA chemistries (2′,4′-BNA/LNA and amido-bridged nucleic acid [AmNA]). Each of these ASOs was 14 nucleotides long (Figure 1A). All ASOs were designed to target the coding region of the human *PCSK9* mRNA (2,079 bp). The library of ASOs corresponded to sequences that ranged across the target transcript’s coding region. The *in vitro* screen was performed with the CEM method on Huh-7, a human hepatoma cell line. The final concentrations of ASOs and CaCl_2_ in the screening cultures were 200 nM and 9 mM, respectively. The initial screen revealed that the level of knockdown activity varied considerably among the 180 ASOs tested here (Figure 1B). The screen also found that there was a strong correlation between the knockdown activity of 2′,4′-BNA/LNA-modified ASOs and their AmNA-modified counterparts (Figure 1C). We selected several ASOs that showed high activity in the first round of screening and subjected these molecules to rescreening in a second round, with the goal of confirming potential candidates (Figures 1D and 1E). Sequence-scrambled LNA gapmer controls showed no knockdown of the target, whether constructed with or without GalNAc. Through a series of *in vitro* assays, HsPCSK9-311-AM, HsPCSK9-1811-AM, and HsPCSK9-1811-LNA (where the AM and LNA suffixes indicate AmNA and 2′,4′-BNA/LNA chemistries, respectively) were identified. Both the 311 and the 1811 series are apparently very potent; however, the 311 series lacks homology with rat sequences, which would make the 1811 series more attractive as candidates for clinical drug development. The melting temperatures (*T*_m_s) against their complementary RNAs and 50% inhibitory concentration (IC_50_) values of these selected candidates, along with those of SPC5001, a known PCSK9 inhibitor, were determined and are provided in Table 1. HsPCSK9-311-AM(14) and -LNA(14) showed very similar *T*_m_ values of ∼73°C and IC_50_ values of ∼10–11 nM. Although the *T*_m_ values of the HsPCSK9-1811 series are ∼10°C lower than those of the HsPCSK9-311 series, the HsPCSK9-1811 series showed higher potency. In this series, HsPCSK9-1811-LNA(14) has a *T*_m_ that is slightly (+3°C) higher than that of HsPCSK9-1811-AM(14), and the former also exhibited slightly higher potency. Santaris’s SPC5001 14-mer showed the lowest *T*_m_; nevertheless, as expected, SPC5001 exhibited excellent potency (IC_50_ = 8.9 nM). The 2-9-2-1 gapmer system was selected based on previous studies by ourselves and others showing that 14-mer gapmers generally provide affinities sufficient for binding to their target RNAs and exhibit activity both *in vitro* and *in vivo.*^27^^,^^28^ Thus, our CEM-based *in vitro* screening successfully identified excellent candidates with activities that were at least comparable to that of SPC5001; we therefore decided to proceed to further *in vivo* validation.

**Table 1:** Properties of candidate ASOs selected from CEM *in vitro* screening

Entry	ID	Sequence^a^	*T*_m_ (°C)^b^	IC_50_ (nM)
1	HsPCSK9-311-AM(14)	GAggtatccccGGc	73 ± 0.1	10.4
2	HsPCSK9-311-LNA(14)	GAggtatccccGGc	73 ± 0.4	11.1
3	HsPCSK9-1811-AM(14)	G5attccagac5Tg	63 ± 0.1	9.8
4	HsPCSK9-1811-LNA(14)	G5attccagac5Tg	66 ± 0.2	7.4
5	SPC5001 (LNA modification)	TG5tacaaaac55A	60 ± 0.2	8.9
6	AZD8233 (cEt modification)	AATaatctcatgt5AG	NA	NA

CEM, Ca^2+^ enrichment of medium.

a Upper and lower cases indicate BNA (LNA, AmNA, and cEt) and native DNA (underline is 5-methyl cytidine), respectively. AM, AmNA, LNA, 2′,4′-BNA/LNA; 5, 5-methyl cytidine BNAs. All internucleotide linkages are phosphorothioate.

b Absorbance versus temperature was measured at 260 nm in 100 mM NaCl, 10 mM sodium phosphate, 0.1 mM EDTA, pH 7.0. The concentration of ASO/RNA is 1.0 μM. Data are presented as mean values ± SD. NA, not available.

### Evaluation in monkeys of efficacy of ASOs selected through a set of *in vitro* challenges

**Figure 2:**
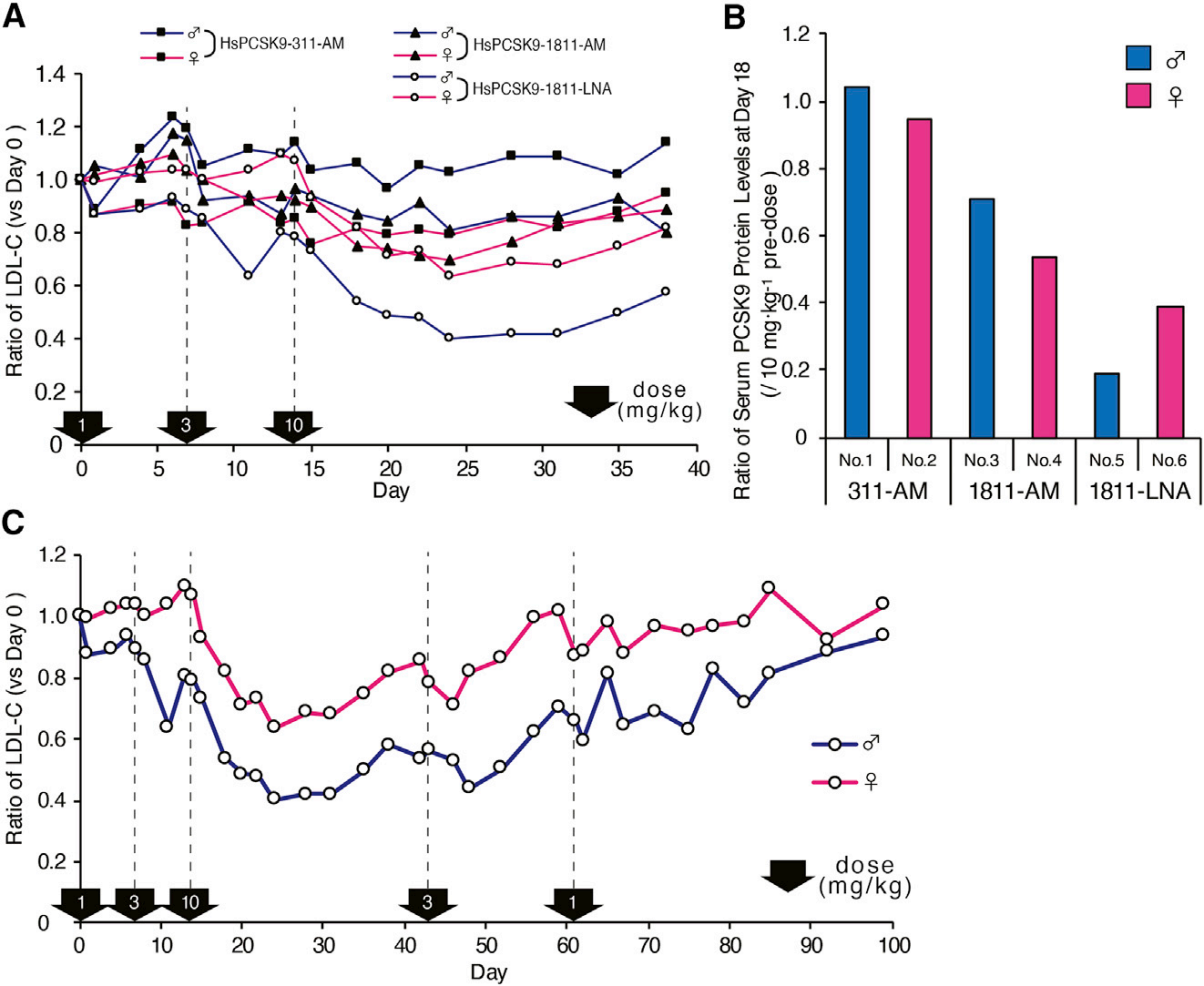
*In vivo* screening of ASOs identified by *in vitro* screening (A) Serum LDL-C-lowering effects of ASOs. Treatment started with one subcutaneous dose at 1 mg/kg, followed by subcutaneous doses at 3 and 10 mg/kg on days 7 and 14, respectively, as indicated by the arrows along the x axis. The y axis indicates the ratio of serum LDL-C levels normalized to the pre-dose (day 0) baseline. (B) Change ratio of serum PCSK9 protein levels on day 18 (normalized to those observed prior to dosing at 10 mg/kg). (C) Ratio of serum LDL-C levels in monkeys treated with HsPCSK9-1811-LNA (normalized to the pre-dose [day 0] baseline). After administration at 10 mg/kg, HsPCSK9-1811-LNA was injected at doses of 3 and 1 mg/kg on days 42 and 61, respectively, as indicated by the vertical arrows along the x axis.

The ASOs (HsPCSK9-311-AM, HsPCSK9-1811-AM, and HsPCSK9-1811-LNA) selected through the *in vitro* screen were evaluated in a dose-escalation study with cynomolgus monkeys. Each ASO was administered subcutaneously once weekly for 3 weeks at doses of 1, 3, and 10 mg/kg in individual male and female monkeys. Serum low-density lipoprotein cholesterol (LDL-C) levels were measured over time through 38 days post-injection. Among the three candidates, HsPCSK9-1811-LNA provided the largest attenuation of cholesterol levels, achieving a nadir of an ∼60% decrease in LDL-C on day 24, after the third injection at 10 mg/kg (Figure 2A). As with LDL-C, serum PCSK9 protein levels fell to the lowest levels (80% of the pre-injection level) after dosing with HsPCSK9-1811-LNA (Figure 2B).

Next, we tested the dose responsiveness and the possible recovery of LDL-C levels following dosing with the most potent candidate, HsPCSK9-1811-LNA. Additional doses of 3 and 1 mg/kg of HsPCSK9-1811-LNA were administered on days 42 and 61, respectively, to animals originally dosed at 1, 3, or 10 mg/kg. As seen in Figure 2C, about a week after the fourth injection of 3 mg/kg on day 42, a significant reduction of LDL-C level was observed reproducibly; in contrast, only a limited effect on LDL-C was seen following the day 61 dose at 1 mg/kg, as we expected. A complete recovery of LDL-C to the baseline level was observed at 100 days after the first injection.

### Evaluation of the safety of HsPCSK9-1811-LNA in rat and monkey

**Figure 3:**
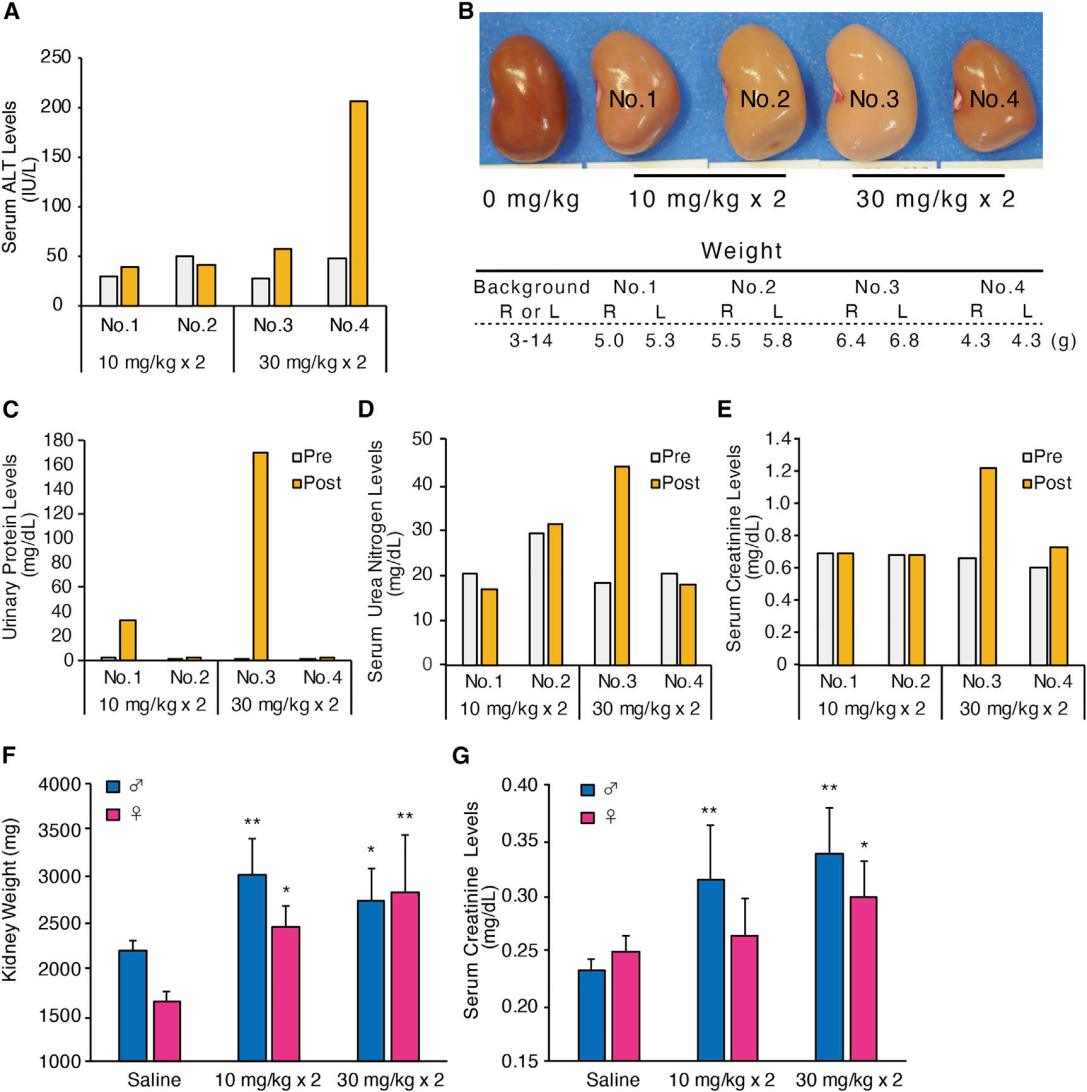
Safety studies of HsPCSK9-1811-LNA in monkey and rat (A–E) HsPCSK9-1811-LNA was injected subcutaneously in monkeys at 10 or 30 mg/kg/week (n = 2). Two weeks after the first dose, selected biochemical parameters were analyzed. (A) Serum ALT levels of the monkeys. (B) Kidneys of HsPCSK9-1811-LNA-dosed monkeys or a non-dosed monkey. R and L represent the weight of the right and left kidneys, respectively. (C) Urinary protein levels at baseline or post-dose. (D) Serum urea nitrogen levels at baseline or post-dose. (E) Serum creatinine levels at baseline or post-dose. (F and G) HsPCSK9-1811-LNA was injected subcutaneously in rats at 10 or 30 mg/kg/week; both male (n = 5) and female (n = 5) rats were used. Two weeks after the first dose, rats were euthanized, and selected biochemical parameters were analyzed. (F) Weights of the kidneys. (G) Serum creatinine levels. Data are presented as mean + SD. p values (versus Saline group) were determined by Dunnett’s multiple comparison tests.∗p < 0.05, ∗∗p < 0.01. Bars having no symbol were not significant.

HsPCSK9-1811-LNA was shown to have a strong LDL-C-lowering effect. To further validate the molecule as an appropriate clinical candidate, we performed 2-week safety studies for HsPCSK9-1811-LNA in rat and monkey. First, groups of two monkeys each were injected subcutaneously, once weekly for 2 weeks, with 10 or 30 mg/kg HsPCSK9-1811-LNA. The monkeys were euthanized 1 week after the final injection. Serum alanine aminotransferase (ALT) levels were elevated in one of the two monkeys treated with 30 mg/kg of the ASO (Figure 3A). As we expected, discoloration and enlargement of the kidneys were observed in all the animals (kidney weights are provided in Figure 3B for reference), and elevation of urine protein levels, serum creatinine, and urea nitrogen also were seen in some animals (Figures 3B–3E). Histopathological examination revealed mild injury with degeneration, karyomegaly, single-cell necrosis, and vacuolation in proximal tubules in both animals dosed at 30 mg/kg. Similar but milder effects also were observed in the monkeys dosed at 10 mg/kg. On the other hand, only moderate histopathological findings were observed in the liver.

In rat experiments, groups of 5 animals each were injected subcutaneously with 10, 30, or 100 mg/kg HsPCSK9-1811-LNA. Four of five male rats and one of five female rats administered 100 mg/kg ASO were found dead within 1 week; the remaining rats were euthanized for humane reasons. The rats of the 10- and 30-mg/kg dose groups were administered a second dose after 1 week and observed for another week before euthanasia, necropsy, and collection of tissues for histopathological evaluation. As in the monkey study, an enlargement of the kidneys and an increase of serum creatinine levels were observed (Figures 3F and 3G). Several foci of tubular injury also were observed in the histopathological analysis, and the degree of renal injury in rat appeared more severe than in monkey at both dose levels.

### Evaluation of GalNAc conjugate in monkey

**Figure 4:**
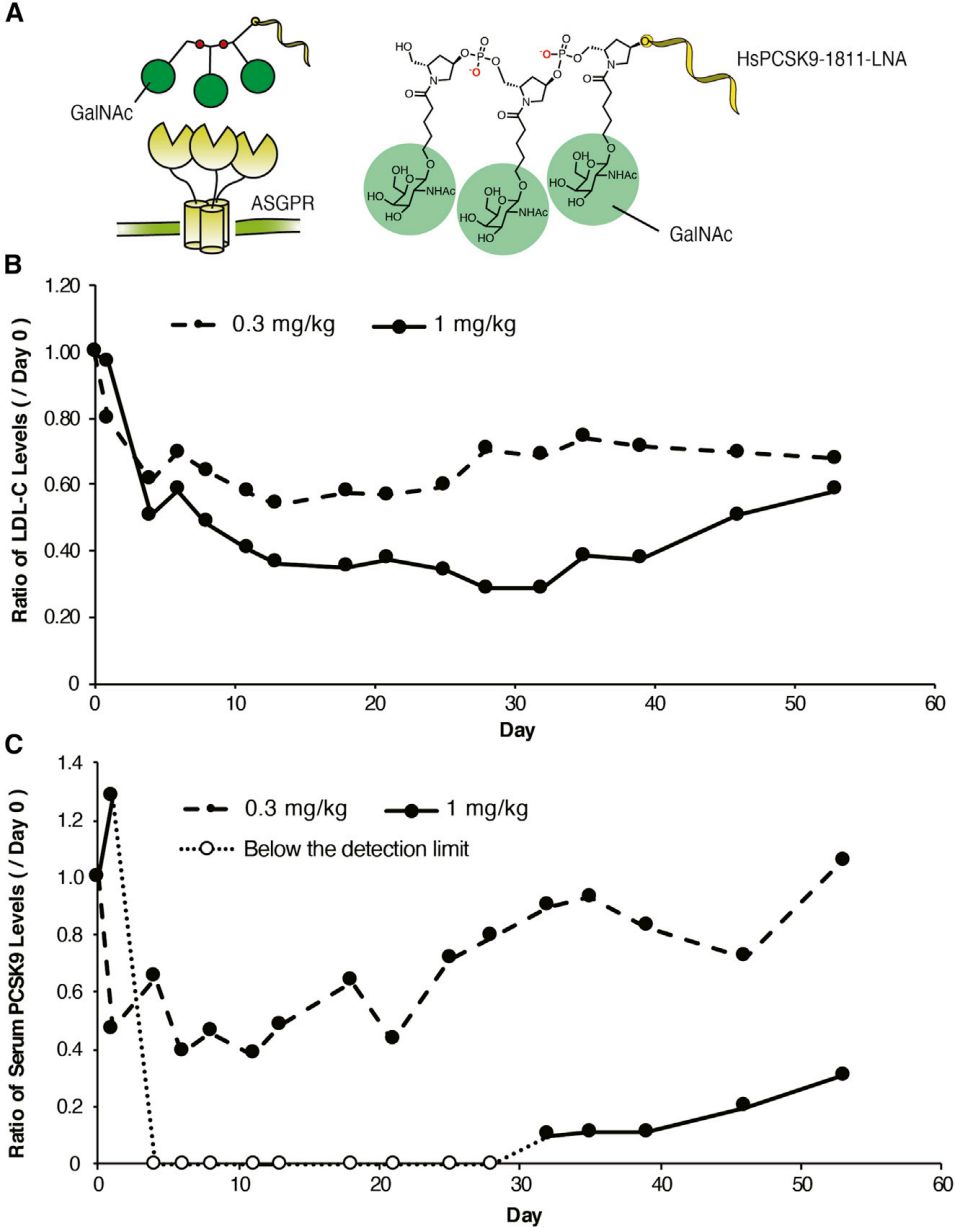
Evaluating the efficacy of GalNAc-conjugated HsPCSK9-1811-LNA in monkey One monkey was administered subcutaneously with *N*-acetylgalactosamine (GalNAc)-conjugated HsPCSK9-1811-LNA (HsPCSK9-1811-LNA-GN) as a single dose at 0.3 or 1 mg/kg. (A) The structure of the GalNAc conjugate. Left: Schematic of recognition of GalNAc-conjugated antisense oligonucleotide (ASO) by asialoglycoprotein receptor (APSGR), facilitating uptake of the ASO. Right: chemical structure of the GalNAc conjugate containing three monomeric GalNAc units (the structure is shown in Figure 1A). (B) Change ratio of serum LDL-C levels normalized to pre-dose (day 0) baseline. (C) Change ratio of serum PCSK9 levels normalized to pre-dose (day 0) baseline. The lower limit of detection is 12 ng/mL.

As we anticipated, based on the previous results with SPC5001, the LNA-based ASO we selected also displayed a relatively narrow therapeutic margin, despite exhibiting a strong LDL-C-lowering effect. To avert the renal burden of this ASO, we next incorporated a GalNAc ligand into the 5′ terminus of HsPCSK9-1811-LNA (Figure 4A), yielding a molecule that we designated HsPCSK9-1811-LNA-GN. For this purpose, we employed a monomer-type GalNAc unit phosphoramidite reagent; we previously reported the synthesis of this reagent and its use in facilitating the introduction of GalNAc into oligonucleotides generated via conventional automated DNA synthesizers.^9^^,^^11^

The potency of HsPCSK9-1811-LNA-GN was evaluated in a single-dose study using monkeys, dosing one monkey each at 0.3 and 1 mg/kg. The dose level of the GalNAc-conjugated ASO was rationalized based on our previous study, in which GalNAc-ASO conjugates were shown to be at least 10 times more potent than their unconjugated counterparts.^9^^,^^16^ After serial collection of serum samples over 53 days post-injection, we analyzed LDL-C and PCSK9 protein levels. Surprisingly, LDL-C levels were considerably decreased at both dose levels, starting only 4 days after the first administration, and the effect was maintained for >50 days, with a maximum reduction of 46% or 71% following dosing at 0.3 or 1 mg/kg, respectively (Figure 4B). Furthermore, serum PCSK9 levels fell below the detection limit of a commercial ELISA system on the fourth day post-dose and remained below this detection limit for >1 month thereafter (Figure 4C). Thirty-five days after administration at 0.3 mg/kg, serum PCSK9 concentrations returned to the pre-dose level.

### Evaluating the safety of the GalNAc conjugate in rat

**Figure 5:**
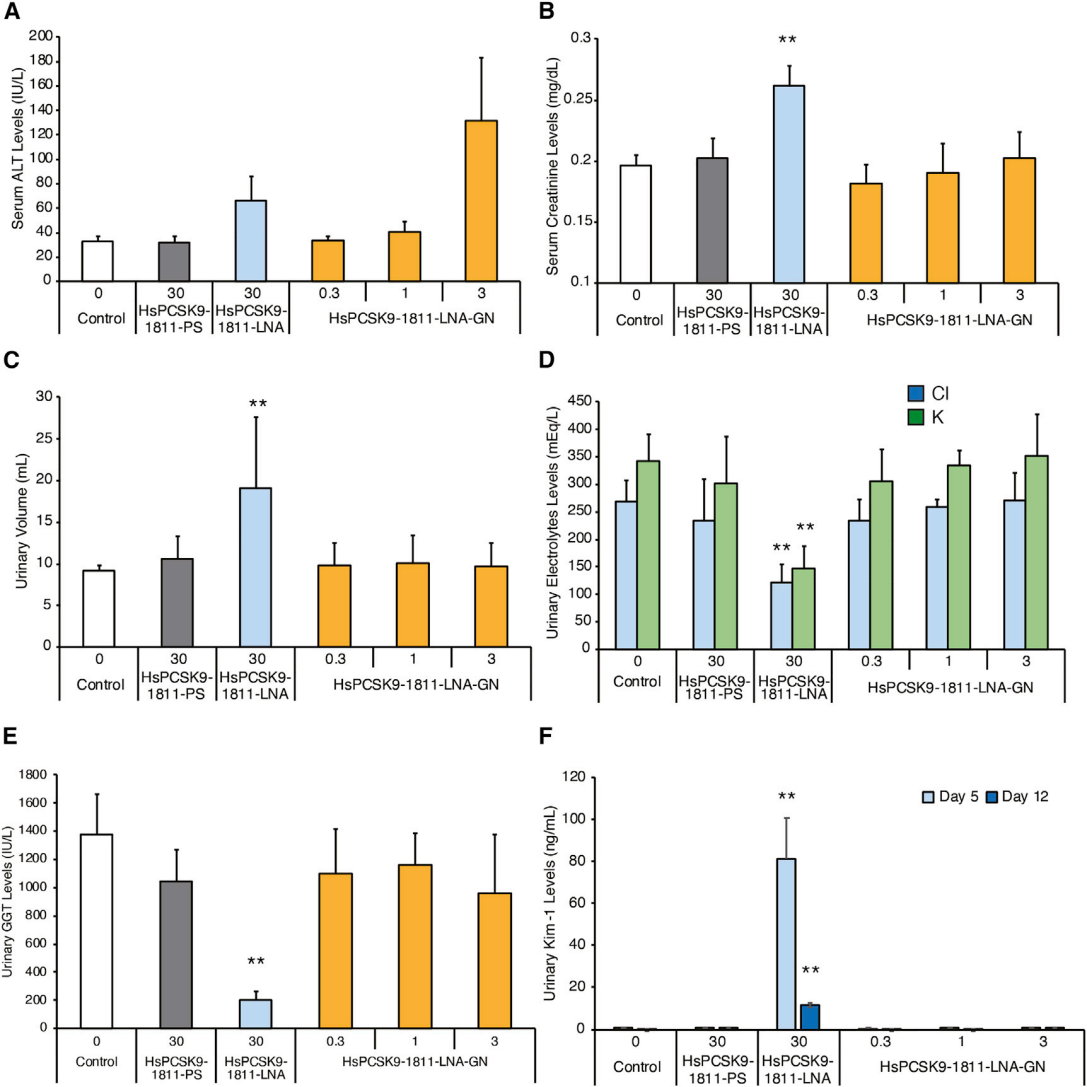
Safety study of HsPCSK9-1811-PS, HsPCSK9-1811-LNA, and HsPCSK9-1811-LNA-GN in male rats ASOs were injected subcutaneously twice weekly for 2 weeks (two doses total) in rats (n = 5). Both HsPCSK9-1811-PS and HsPCSK9-1811-LNA were administered (two doses each) at 30 mg/kg/week. HsPCSK9-1811-LNA-GN was administered (in separate groups; two doses each) at 0.3, 1, or 3 mg/kg/week. Urine was collected on days 5 and 12. Two weeks after the first administration, blood samples were collected, animals were euthanized, and liver and kidney tissues were harvested. (A) Serum alanine aminotransferase (ALT) levels on day 14. (B) Serum creatinine levels on day 14. (C) Urinary volume on day 12. (D) Urinary electrolyte levels on day 5. (E) Urinary γ-glutamyltransferase (GGT) levels on day 12. (F) Urinary kidney injury molecule-1 (Kim-1) levels on days 5 and 12. n = 5 for each parameter. Data are presented as mean + SD. p values (versus Control group) were determined by Dunnett’s multiple comparison tests. ∗∗p < 0.01. Bars having no symbol were not significant,

In the previous section, we demonstrated that efficacy of the candidate molecule was improved dramatically by conjugation to GalNAc. To address the effect of our GalNAc approach on kidney toxicity, the conjugate was administered in rat. We performed this experiment using three different ASOs: HsPCSK9-1811-PS, HsPCSK9-1811-LNA, and HsPCSK9-1811-LNA-GN. HsPCSK9-1811-PS is a non-LNA antisense control that has a sequence identical to those of the other two compounds. Whereas HsPCSK9-1811-PS and HsPCSK9-1811-LNA were administered subcutaneously in rat as two once-weekly doses at 30 mg/kg, HsPCSK9-1811-LNA-GN was administered at two dose levels of 0.3 and 3 mg/kg. The 0.3-mg/kg dose was selected based on the 10-fold greater potency of the conjugated molecule (as demonstrated by the effect on serum PCSK9 protein levels in monkey); the 3-mg/kg dose was selected as the higher dosage for HsPCSK9-1811-LNA-GN because we considered a 10-fold margin a reasonably wide therapeutic window in terms of the renal toxicity, based on our previous data and the example of SPC5001. At 2 weeks after the first dose (i.e., 1 week after the 2^nd^ dose), rats dosed with HsPCSK9-1811-LNA exhibited attenuation of body weight gain compared to animals dosed with the other two test compounds. Serum ALT levels were elevated 2- and 4-fold after dosing with 30 mg/kg HsPCSK9-1811-LNA or 3 mg/kg HsPCSK9-1811-LNA-GN, respectively, compared to the level in the saline-treated control (Figure 5A). Serum creatinine levels increased significantly only in the animals dosed with 30 mg/kg HsPCSK9-1811-LNA compared to the level in the control (Figure 5B).

On days 5 and 12, urine samples were collected and analyzed for a range of biochemical and toxicological parameters. The urinary volume increased on days 5 and 12 (Figure 5C), and an associated decrease was seen in the urinary concentration of electrolytes (e.g., potassium and chloride) on day 5 after dosing with HsPCSK9-1811-LNA (Figure 5D). Also, urinary levels of γ-glutamyltransferase (GGT) were decreased significantly by HsPCSK9-1811-LNA administration. Kidney injury molecule-1 (Kim-1) is known as an early indicator of renal proximal tubule injury;^29^ Kim-1 urinary levels were elevated significantly in the HsPCSK9-1811-LNA-treated group, indicating kidney injury (Figures 5E and 5F). Note that Kim-1 levels peaked on day 5 before subsequently declining.

**Figure 6:**
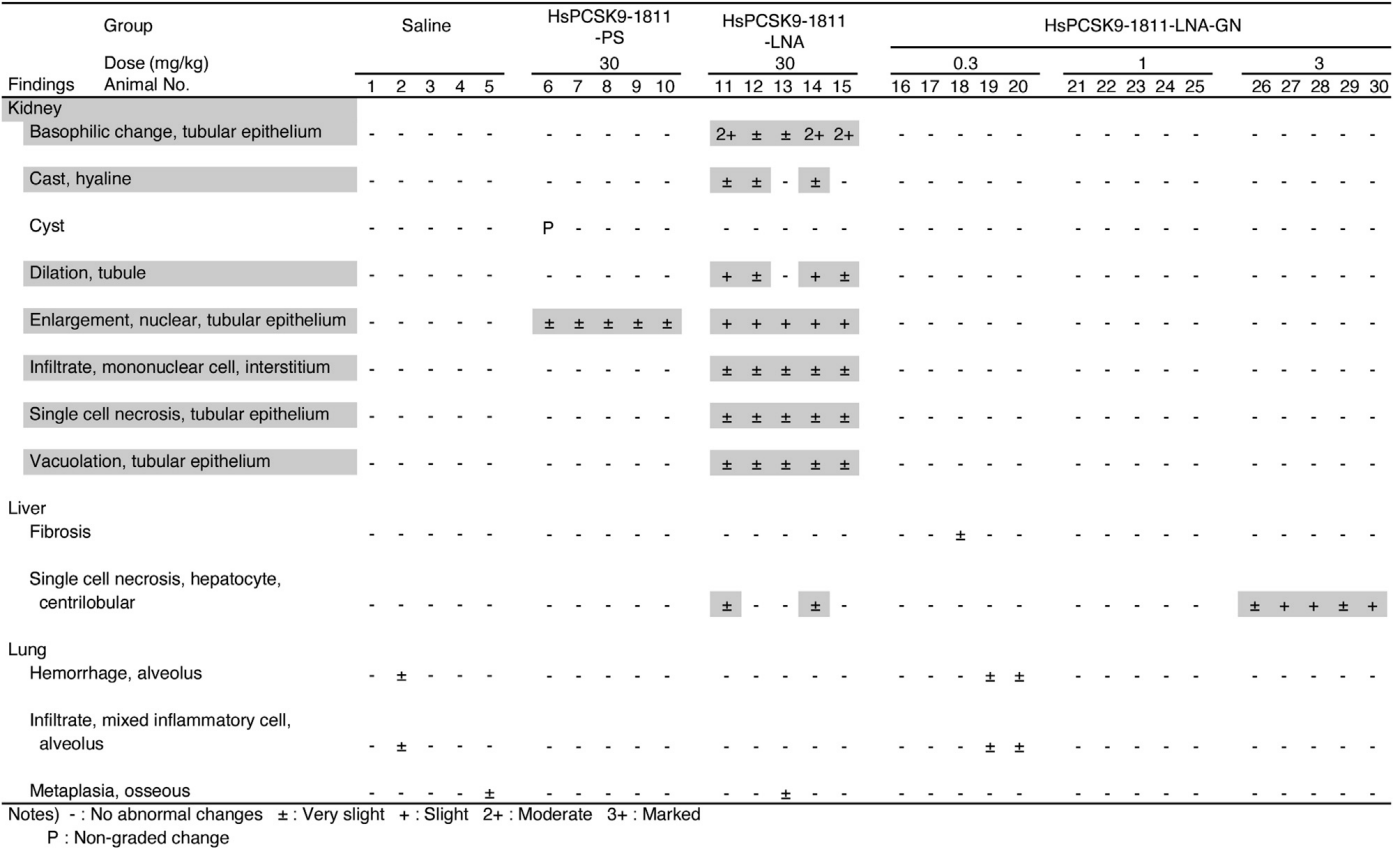
Histopathological analysis of each antisense oligonucleotide-dosed rat

**Figure 7:**
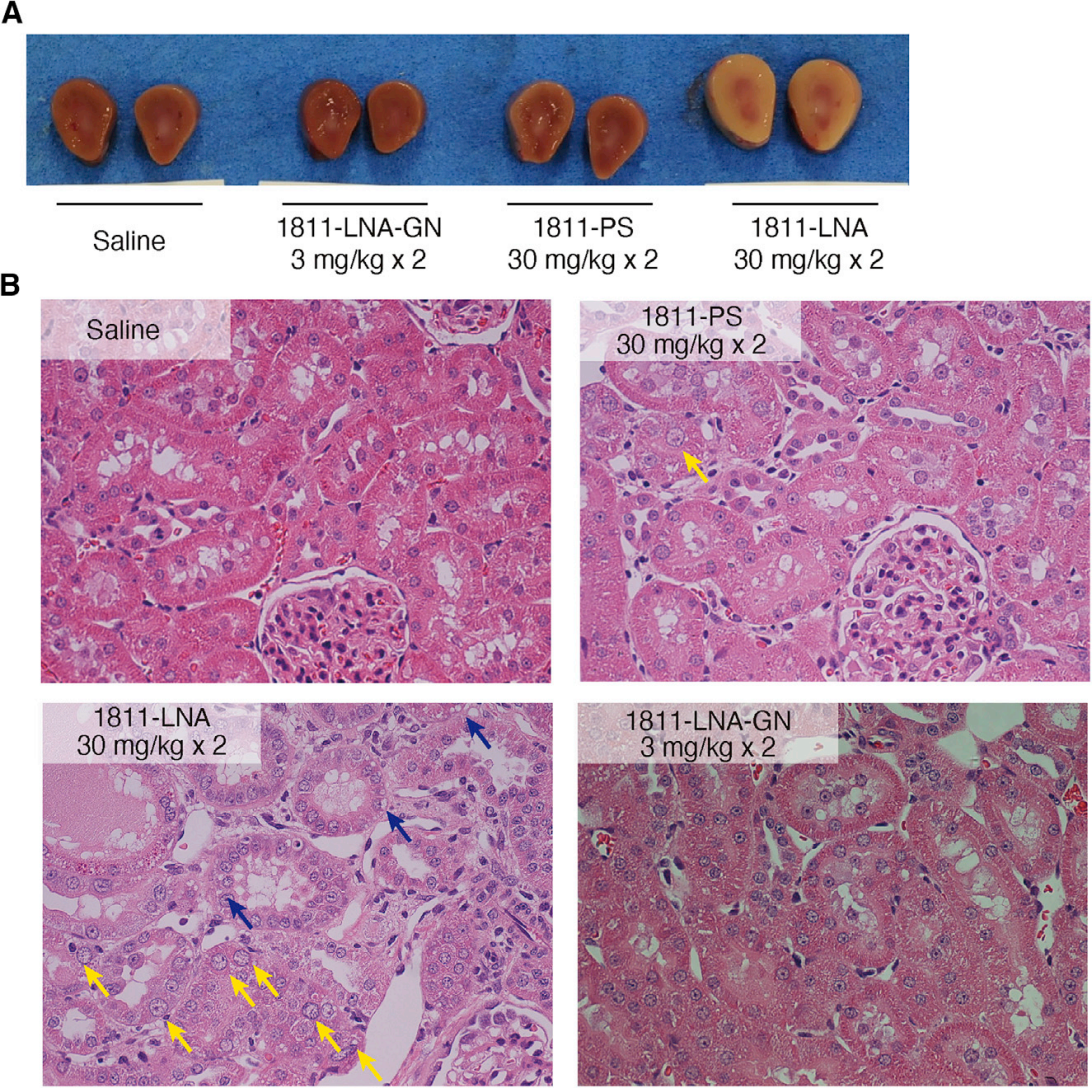
Histopathological analysis of kidneys (A) Gross pathology of the kidneys of HsPCSK9-1811-LNA-dosed or saline-dosed rats. (B) Hematoxylin and eosin (H&E) staining of the sections of fixed kidney (×400 magnification). Yellow arrows indicate nuclear enlargement of tubular epithelium. Blue arrows indicate vacuolation of tubular epithelium.

All rats were euthanized and necropsied on day 14 (1 week after the final dose), and tissues were collected and submitted for histopathological evaluation (Figure 6). Macroscopically, the livers of rats treated with HsPCSK9-1811-LNA or 3 mg/kg of HsPCSK9-1811-LNA-GN were bloated; microscopically, very slight single-cell necrosis of the centrilobular hepatocytes was observed in these livers. As shown in Figure 7A, enlarged, discolored kidneys were observed only in the animals dosed with HsPCSK9-1811-LNA, and the weight of these organs was ∼1.5-fold those in the other dosing group. The histopathological analysis of the sections of kidneys from animals dosed with HsPCSK9-1811-LNA showed basophilic change, nuclear enlargement, single-cell necrosis, vacuolation of the tubular epithelium, and infiltration of mononuclear cells into the interstitium (Figures 6 and 7B). Nuclear enlargement also was observed in the kidneys of rats treated with HsPCSK9-1811-PS. In contrast, no change was found in the kidneys of rats treated with either dose level of HsPCSK9-1811-LNA-GN.

## Discussion

In this study, we successfully demonstrated the benefit of using our drug discovery platform and obtained *in vivo*-active ASOs with alleviated “class side effects.” CEM is a simple, highly versatile, and high-throughput *in vitro* screening system that expedites an otherwise painful *in vitro* screening step to facilitate the identification of ASOs with *in vivo* activity. It is known that most cell lines have lost their nucleic acid uptake capacity, impeding our ability to reproduce faithfully the *in vivo* environment, i.e., the situation whereby ASOs are taken up by cells, in the *in vitro* cell culture system. Until now, methods using primary cultured cells or attempts to construct new strains based on primary cells have been used, but these specialized methods are difficult to implement in human cell lines, limiting the development of translational nucleic acid drugs. In the CEM method, on the other hand, calcium ions stimulate intracellular uptake by facilitating aggregation of serum albumin, which in turn acts on the cultured cells.^2^ Unlike conventional transfection methods, CEM has proven to better predict the *in vivo* activity of ASOs, enabling the direct exploration of ASOs corresponding to the human *PCSK9* sequence and bypassing experiments in small rodents. Notably, CEM does not limit the chemistry or physicochemical properties of ASOs of interest. We used 2′,4′-BNA/LNA- and AmNA-based ASOs, the chemistry of which helps to fortify binding affinity, Of several potent inhibitors selected using CEM, all of the tested candidates demonstrated attenuation of PCSK9 expression in non-human primates. The selected 14-mer gapmers (using the 2-9-2-1 system) have been shown to have affinities sufficient to provide knockdown of PCSK9 activity both *in vitro* and *in vivo* owing to the high-affinity BNA modification (Table 1). It appears that binding affinity depends more on sequence than on strand length or the number of LNA units for ASOs in this length range (13- to 16-mers). In a biodistribution study of LNA gapmers of different lengths, Straarup et al.^27^ detected no significant difference in liver and kidney localization of 16-mers versus 12-mers; moreover, in general, the more PS bonding, the higher potential for tissue toxicity,^30^ BNA gapmers often are ∼13–16 units in length. In addition, it is known that shorter-chain ASOs generated through metabolism and digestion are eliminated preferentially, and longer-chain ASOs accumulate in tissues for a longer period of time, contributing to side effects.^31^ On the basis of these findings, we opted to employ the 2-9-2-1 14-mer format of gapmers in the present work.

On the other hand, we should note that CEM failed to provide high-accuracy differentiation in activity between the two chemical modifications (i.e., 2′,4′-BNA/LNA versus AmNA). In a previous study, Uehara et al. demonstrated the promising effect of AmNA-based gapmer ASOs in a neurodegenerative disease context.^32^ However, a major difference between the work of Uehara et al. and ourselves was that in Uehara’s work AmNA ASOs were delivered locally to the mouse brain through intracerebroventricular injection. CEM’s inability to distinguish the chemistries of BNAs presumably derives from the fact that the different chemical modifications have distinct effects on pharmacokinetic properties such as protein-binding affinity, membrane permeability, and blood and intracellular stability. Clearly, further work will be needed to analyze the pharmacological or pharmacokinetic property differences between ASOs employing AmNA and 2′,4′-BNA/LNA modifications.

ASOs are eliminated primarily in the kidneys via proximal tubular epithelial cells.^5^ The excess accumulation of ASOs in such cells induces osmotic imbalance, which may explain the nephrotoxicity of this class of compounds. In this context, we have demonstrated that the introduction of GalNAc counteracts the accumulation of ASOs in the kidneys, resulting in the complete elimination of kidney injury and securing an ∼10-fold safety margin.^3^^,^^33^

Although there have been many reports on the hepatotoxicity issues of ASOs in early drug development, the pre-clinically observed nephrotoxicity of 2′,4′-BNA/LNA gapmers such as SPC5001 was our primary target in this study. Notably, we have shown an effective approach to avoid nephrotoxicity using GalNAc conjugation, which enables more drug in liver and less in kidney. At the same time, we are aware that it is indeed necessary to be able to select ASO drugs with a wider safety margin for the treatment of chronic diseases such as dyslipidemia. Recently, Dieckmann et al.,^34^ Shen et al.,^35^ and Papargyri et al.^36^ have developed or utilized an *in vitro* method, the measurement of cellular caspase 3/7 activity, to evaluate the hepatotoxicity of BNA gapmer ASOs in mice. If used in combination with our CEM screening strategy, such a method may permit estimation not only of hepatotoxicity in mice but also of other toxicities in non-human primates and humans.

Additionally, it has been reported that ASOs conjugated with triantennary-type GalNAc have lower nephrotoxic potential than non-conjugates in *in vitro* experiments, as assessed with renal proximal tubule epithelial cells.^37^ Hence, the GalNAc conjugation strategy may have advantages for reducing the renal drug burden, not only by improving the pharmacokinetics of ASOs but also as a result of the chemical properties of GalNAc. Further experiments will be needed to clarify whether the effect on renal proximal tubule epithelial cells is also obtained with the monovalent-type GalNAc used in the present experiments. It also has been reported that small structural perturbations can change the hepatotoxicity profile of BNA gapmers.^35^ Therefore, it may be beneficial to perform a systematic evaluation of the potential positive effects on toxicity of our monomeric GalNAc conjugation.

A moderate increase of serum ALT was observed for the highest dose of HsPCSK9-1811-LNA-GN in rats. Hepatotoxicity associated with ASOs is observed more frequently in rodents, and recent studies suggest that its onset can be ascribed to an interaction with RNase H or intracellular proteins.^35^^,^^38^^,^^39^ In a previous study, we showed that GalNAc also helps to reduce hepatotoxicity in mice, most likely by facilitating the selective accumulation of ASOs in the parenchymal hepatocytes. There also is potential difficulty in setting the appropriate dose range for toxicity testing in rodents based on the activity in monkeys, especially when testing ASOs for which there is no sequence homology between rodents and primates. Extensive further investigation of dose-toxicity correlation needs to be conducted in rodents and non-human primates. At the same time, optimization of ASO sequences will need to be performed based on rational strategies to reduce potential hepatotoxicity in rodents; approaches such as those suggested by Shen et al. and Migawa et al. are expected ameliorate the situation.

Patients with *PCSK9* loss-of-function mutations have a much lower rate of CVD than non-carriers.^40^ Because ASOs prevent production of the PCSK9 protein itself and can mimic the pathophysiology of individuals with *PCSK9* loss-of-function mutations, the ASOs obtained in the present study are expected to exhibit increased potency for reducing the risk of CVD compared with other types of inhibitors (like antibodies) that are in current use.

ASOs with chemical modifications hold great promise for therapeutic application if such molecules are effective and safe. Here, we established a robust drug discovery scheme and successfully identified highly potent and safe ASOs that showed activity against human *PCSK9* mRNA while possessing a liver-targeting mechanism. In particular, our HsPCSK9-1811-LNA-GN did show comparable potency and efficacy in non-human primates to an anti-PCSK9 cEt gapmer with a triantennary GalNAc unit, AZD8233, which showed great potential in human trials.^41^

One limitation of this study is that the bare minimum number of animals was used in the monkey studies because of the limited number of animals that were available. Although it is reasonable from the perspective of animal protection, and a clear dose dependency was confirmed in the present study, additional follow-up studies with certain control ASOs will be needed to support the alleged efficacy and safety of the ASOs; such studies currently are being conducted as part of pre-clinical studies. Nevertheless, we believe that this approach may be generalizable to the isolation of clinically relevant ASOs, regardless of the target.

## Materials and methods

### Oligonucleotides

The ASOs used in this work were synthesized by Gene Design (Osaka, Japan). The phosphoramidite monomers of AmNA and the monovalent GalNAc units were obtained as previously described.^9^^,^^42^ The sequences of the ASOs targeting *PCSK9* mRNA are shown in Tables S1 and S2. Notably, the ASO (HsPCSK9-1811) sequence used in rat and monkey studies was complementary to the *PCSK9* mRNA from both rat and monkey.

### *In vitro* screening

Huh-7 cells, a human hepatoma cell line, were maintained at 37°C and 5% CO_2_ in Dulbecco’s modified Eagle’s medium (DMEM; Sigma-Aldrich, St. Louis, MO, USA) supplemented with 10% heat-inactivated fetal bovine serum (FBS; Thermo Fisher Scientific, Waltham, MA, USA) and 1% (v/v) penicillin-streptomycin (Thermo Fisher Scientific). Huh-7 cells were seeded at 5,000 cells/well in 96-well plates (Corning, New York, NY, USA). Twenty-four hours after seeding, ASOs were added in 10% FBS-DMEM containing 9 mM CaCl_2_. After another 24 h, cDNA was synthesized directly from cell lysates with a SuperPrep Cell Lysis & RT Kit for qPCR (Toyobo, Osaka, Japan). Quantitative reverse transcriptase-polymerase chain reaction (qRT-PCR) was performed with a TaqMan Fast Advanced Master Mix (Thermo Fisher Scientific) and analyzed with a StepOnePlus system (Applied Biosystems, Foster City, CA, USA). For the IC_50_ experiments, HsPCSK9-311-AM(14), HsPCSK9-311-LNA(14), HsPCSK9-1811-AM(14), HsPCSK9-1811-LNA(14), and SPC5001 were each transfected into Huh-7 cells at 0.8–200 nM (8–9 points) with the above CaCl_2_-containing medium. The experiments were performed in technical quadruplicate and were repeated three times. IC_50_s were calculated by the log(inhibitor) versus response (three parameters) method in Prism 9.2.0 (GraphPad). The TaqMan probes (Applied Biosystems) used in this assay were as follows:

Human PCSK9: Hs00545399_m1 (FAM)
Human GAPDH: Hs02758991_g1 (VIC)

### Melting temperature experiments

UV melting experiments were performed on a Shimadzu UV-1850 spectrophotometer equipped with the TMSPC-8 *T*_m_ analysis system (Shimadzu, Kyoto, Japan). Two complementary single-stranded oligonucleotides were mixed in a 10 mM sodium phosphate buffer (pH 7.0) containing 100 mM NaCl to give a 1.0 μM oligonucleotide solution. The mixture was annealed by heating at 95°C for 3 min and then cooled at −1°C/min to 20°C. *T*_m_ was estimated with the software provided with the TMSPC-8 and defined as the temperature at which the formed duplexes were half-dissociated. The temperature-dependent change in the optical absorption was recorded at 260 nm, and the scan rate was 0.5°C/min from 4°C to 94°C. Each averaged *T*_m_ value was determined as the intersection point between the absorption on temperature and the median between the two baselines from three independent measurements.

### Animals and conditions

The animal studies using rats were conducted at a certified contract organization (Shin Nippon Biomedical Laboratories, Kagoshima, Japan) that is fully accredited by AAALAC International. These studies were approved by an Institutional Animal Care and Use Committee (Approval Nos. IACUC864-007 and IACUC864-012) and were performed in accordance with the animal welfare bylaws of Shin Nippon Biomedical Laboratories Drug Safety Research Laboratories. Rats were maintained on a 12-h light/12-h dark cycle (lights on from 7:00 AM to 7:00 PM) and provided with *ad libitum* access to a standard chow (CRF-1; Oriental Yeast, Tokyo, Japan) and water.

The non-human primate studies were also conducted at Shin Nippon Biomedical Laboratories. These studies were approved by an Institutional Animal Care and Use Committee (Approval Nos. IACUC864-004, IACUC864-009, and IACUC864-010) and were performed in accordance with the animal welfare bylaws of Shin Nippon Biomedical Laboratories Drug Safety Research Laboratories. The studies were performed as non-Good Laboratory Practice (GLP) experiments in male or female cynomolgus monkeys. The monkey age at initiation of treatment was 2–4 years. The monkeys were housed under a 12-h light/12-h dark cycle (lights on from 7:00 AM to 7:00 PM) and fed once daily with ∼100 g of solid food (HF Primate J 12G 5K9J; Purina Mills, St. Louis, MO, USA); for enrichment, animals were provided with apple slices 5 or more times per week. After dosing or collection of blood, the monkeys were provided with raisins or peanuts as treats.

### *In vivo* screening using monkeys

A weekly dose-escalation study of HsPCSK9-311-AM, HsPCSK9-1811-AM, and HsPCSK9-1811-LNA was used to evaluate the efficacies of these ASOs. Monkeys that had relatively high LDL-C levels were selected from the monkeys at Shin Nippon Biomedical Laboratories. The candidate ASOs were administered subcutaneously to the monkeys (n = 2; one male and one female) at a starting dose of 1 mg/kg (on day 0), followed by 3 mg/kg on day 7 and 10 mg/kg on day 14. Blood samples were collected on days 0 (before dose administration), 1, 4, 6, 7 (before dose administration), 8, 11, 13, 14 (before dose administration), 15, 18, 20, 22, 24, 28, 31, 35, and 38. Monkeys treated with HsPCSK9-1811-LNA received an additional dose of 3 mg/kg on day 42 (i.e., 28 days after the 10 mg/kg dose) and a final dose of 1 mg/kg on day 61. For animals administered the additional doses, additional blood samples were collected on days 42 (before dose administration), 46, 48, 52, 56, 59, 61 (before dose administration), 65, 67, 71, 75, 78, 82, 85, 92, and 99.

### Safety tests using rats

For all rat studies, animals were 6 weeks old at the initiation of dosing. Each ASO was administered to male or female rats (n = 5) by subcutaneous injection at 0.3, 1, 3, 10, 30, or 100 mg/kg or an equivalent volume of 0.9% saline for a control group (n = 5); doses were administered once weekly for 2 weeks (for a total of two doses). Urine was collected on days 5 and 12. Fourteen days after the first administration, blood samples were collected via the inferior vena cava, animals were euthanized, and liver and kidney tissues were harvested. After clotting, the blood samples were centrifuged at 1,700 × *g* for 10 min, and the resulting serum supernatants were stored at −80°C until analysis. Liver and kidney tissues were fixed in 10% neutral buffered formalin.

### Safety tests using monkeys

To evaluate the safety of HsPCSK9-1811-LNA in monkey, a 2-week study was conducted in the same manner as the safety test using rats. HsPCSK9-1811-LNA was administered subcutaneously in monkeys (n = 2) at 10 or 30 mg/kg, once weekly for 2 weeks (for a total of 2 doses). Urine was collected on days 2, 6, 9, 13, and 14. Blood samples were collected on days −2, 3, 7, 10, and 14; serum was isolated and stored as described above. Fourteen days after the first administration, animals were euthanized, and liver and kidney tissues were harvested and fixed in 10% neutral buffered formalin.

### Evaluation of the efficacy of HsPCSK9-1811-LNA-GN in monkey

To evaluate the efficacy of HsPCSK9-1811-LNA-GN in monkey, a single-dose study was conducted. One monkey was administered subcutaneously with HsPCSK9-1811-LNA-GN at a single dose of 0.3 or 1 mg/kg. After the first administration, blood samples were collected on days 1, 4, 6, 8, 11, 13, 18, 21, 25, 28, 32, 35, 39, 46, and 53; serum was isolated and stored as described above.

### Blood chemistry analysis and urinalysis

Serum ALT, aspartate aminotransferase (AST), creatinine, urea nitrogen, total cholesterol, LDL-C, urinary GGT, and protein levels were measured with an automated analyzer (JCA-BM6070; JEOL, Tokyo, Japan). Serum PCSK9 protein levels were determined with the Human PCSK9 Quantikine ELISA Kit (R&D Systems, Minneapolis, MN, USA). Urinary Kim-1 levels were determined with xMAP technology using a Bio-Plex 200 Systems (Bio-Rad) kit according to the manufacturer’s instructions.

### Histopathological analysis

The formalin-fixed tissues were embedded in paraffin, sectioned, and stained with hematoxylin and eosin (H&E). The histopathological analyses were conducted at Shin Nippon Biomedical Laboratories. The scores were classified into five grades according to the histopathological findings.

### Statistical analysis

*In vitro* dose-dependent test and rat study data are presented as mean ± standard deviation (SD). To compare the efficacy of LNA- and AmNA-modified ASOs, Pearson’s correlation coefficients (r value) were calculated, and single linear regression analysis was performed. Statistical significance (p < 0.05) was determined by Dunnett’s multiple comparison tests. Pearson’s correlation coefficients and statistical significance were analyzed with the statistical programming language R.

### Data availability

Data supporting the findings of this study are available from the corresponding author upon reasonable request.

## Supplemental information

Document 1. Tables S1 and S2

Document 2. Article plus supplemental information
