# Supplementary material for: Drug discovery and development scheme for liver-targeting bridged nucleic acid antisense oligonucleotides
Source: Mol Ther Nucleic Acids. 2021 Oct 19;26:957–69. doi: 10.1016/j.omtn.2021.10.008 (PMC8560717; doi:10.1016/j.omtn.2021.10.008)
Supplement: Document 1. Tables S1 and S2 [file mmc1.pdf]

OMTN, Volume *26*

Supplemental information

Drug discovery and development scheme for liver-targeting bridged nucleic acid antisense oligonucleotides

Fumito Wada, Tsuyoshi Yamamoto, Tadayuki Kobayashi, Keisuke Tachibana, Kosuke Ramon Ito, Mayumi Hamasaki, Yukina Kayaba, Chisato Terada, Asako Yamayoshi, Satoshi Obika, and Mariko Harada-Shiba

Table S1 Sequences of AmNA-modified ASO

ID	No.	Sequences	ID	No.	Sequences	ID	No.	Sequences
HsPCSK9-11-AM(14)	1	CCgcctggagcTGa	HsPCSK9-821-AM(14)	31	CTggaccagctGGc	HsPCSK9-1381-AM(14)	61	GAgtgtgctgaCCa
HsPCSK9-21-AM(14)	2	GCcaccaggacCGc	HsPCSK9-871-AM(14)	32	CGgctgtacccACc	HsPCSK9-1441-AM(14)	62	CTcagcagctcCTc
HsPCSK9-61-AM(14)	3	GGacccaggagCAg	HsPCSK9-961-AM(14)	33	TAgaggcaggcATc	HsPCSK9-1451-AM(14)	63	ACtggagcagcTCa
HsPCSK9-121-AM(14)	4	AAggctagcacCAg	HsPCSK9-971-AM(14)	34	GGctggggagtAGa	HsPCSK9-1461-AM(14)	64	TCctggagaaaCTg
HsPCSK9-181-AM(14)	5	TGgaaggtggcTGt	HsPCSK9-991-AM(14)	35	GTgatgacctcGGg	HsPCSK9-1471-AM(14)	65	CGcttcccactCCt
HsPCSK9-201-AM(14)	6	ACggatccttgGCg	HsPCSK9-1001-AM(14)	36	GGccccaactgTGa	HsPCSK9-1501-AM(14)	66	CCcccttgggcCTc
HsPCSK9-241-AM(14)	7	TCctccttcagCAc	HsPCSK9-1011-AM(14)	37	GGgcattggtgGCc	HsPCSK9-1541-AM(14)	67	CTcacccccaaAAg
HsPCSK9-311-AM(14)	8	GAggtatccccGGc	HsPCSK9-1041-AM(14)	38	CCaaagtccccAGg	HsPCSK9-1551-AM(14)	68	CGtagacacccTCa
HsPCSK9-321-AM(14)	9	GGatcttggtgAGg	HsPCSK9-1051-AM(14)	39	AAgttggtcccCAa	HsPCSK9-1561-AM(14)	69	CTggcaatggcGTa
HsPCSK9-331-AM(14)	10	AAgacatgcagGAt	HsPCSK9-1071-AM(14)	40	AGaggtccacaCAg	HsPCSK9-1571-AM(14)	70	CAggcagcaccTGg
HsPCSK9-351-AM(14)	11	AGccaggaagaAGg	HsPCSK9-1081-AM(14)	41	CCtggggcaaaGAg	HsPCSK9-1581-AM(14)	71	CCtggggtagcAGg
HsPCSK9-361-AM(14)	12	TTcaccaggaaGCc	HsPCSK9-1091-AM(14)	42	GAtgtcctcccCTg	HsPCSK9-1611-AM(14)	72	CTggtggagctGTg
HsPCSK9-371-AM(14)	13	GCcactcatctTCa	HsPCSK9-1101-AM(14)	43	AGgcaccaatgATg	HsPCSK9-1631-AM(14)	73	GGtccccatgcTGg
HsPCSK9-431-AM(14)	14	AGaggagtcctCCt	HsPCSK9-1121-AM(14)	44	GCaggtgctgcAGt	HsPCSK9-1671-AM(14)	74	AGcctgtgaggACg
HsPCSK9-441-AM(14)	15	GGgcaaagacaGAg	HsPCSK9-1131-AM(14)	45	GTgacacaaagCAg	HsPCSK9-1681-AM(14)	75	TGggagctgcaGCc
HsPCSK9-451-AM(14)	16	GGgatgctctgGGc	HsPCSK9-1161-AM(14)	46	CGtgggcagcaGCc	HsPCSK9-1691-AM(14)	76	CAcctcccagtGGg
HsPCSK9-501-AM(14)	17	GGtattcatccGCc	HsPCSK9-1171-AM(14)	47	ATgccagccacGTg	HsPCSK9-1701-AM(14)	77	CAaggtcctccACc
HsPCSK9-531-AM(14)	18	CCtccaccaggCTg	HsPCSK9-1181-AM(14)	48	CAtggctgcaaTGc	HsPCSK9-1711-AM(14)	78	TTgtgggtgccAAg
HsPCSK9-541-AM(14)	19	AGgagatacacCTc	HsPCSK9-1191-AM(14)	49	CAgacagcatcATg	HsPCSK9-1731-AM(14)	79	GTggcctcagcACa
HsPCSK9-551-AM(14)	20	GCtggtgtctaGGa	HsPCSK9-1231-AM(14)	50	AGtctctgcctCAa	HsPCSK9-1771-AM(14)	80	CTggcctccctGTg
HsPCSK9-561-AM(14)	21	CActctgtatgCTg	HsPCSK9-1241-AM(14)	51	GAagtggatcaGTc	HsPCSK9-1791-AM(14)	81	GGcagcaggaaGCg
HsPCSK9-591-AM(14)	22	CCatgaccctgCCc	HsPCSK9-1251-AM(14)	52	CTttggcagagAAg	HsPCSK9-1801-AM(14)	82	CCtggggcatgGCa
HsPCSK9-641-AM(14)	23	TCtgtggaagcGGg	HsPCSK9-1261-AM(14)	53	TTgatgacatcTTt	HsPCSK9-1811-AM(14)	83	GCattccagacCTg
HsPCSK9-651-AM(14)	24	TGctggcctgtCTg	HsPCSK9-1271-AM(14)	54	CCaggcctcatTGa	HsPCSK9-1821-AM(14)	84	CCttgactttgCAt
HsPCSK9-661-AM(14)	25	CTgtcacacttGCt	HsPCSK9-1281-AM(14)	55	CCtcagggaacCAg	HsPCSK9-1831-AM(14)	85	ATtccatgctcCTt
HsPCSK9-681-AM(14)	26	CTgccaggtggGTg	HsPCSK9-1301-AM(14)	56	GGgggtcagtaCCc	HsPCSK9-1851-AM(14)	86	CCtgctcctgaGGg
HsPCSK9-691-AM(14)	27	CTgaccaccccTGc	HsPCSK9-1311-AM(14)	57	CCaccaggttgGGg	HsPCSK9-1911-AM(14)	87	AGgtcccagggAGg
HsPCSK9-721-AM(14)	28	GCacccttggcCAc	HsPCSK9-1331-AM(14)	58	GGtgctgggggGCa	HsPCSK9-1931-AM(14)	88	GTaggcccccaGGa
HsPCSK9-751-AM(14)	29	CAgttgagcacGCg	HsPCSK9-1351-AM(14)	59	AGctgccaaccTGc	HsPCSK9-1991-AM(14)	89	GCtggtgctgcCTg
HsPCSK9-761-AM(14)	30	CTtcccttggcAGt	HsPCSK9-1361-AM(14)	60	CCtgcaaaacaGCt	HsPCSK9-2021-AM(14)	90	GCagatggcaaCGg

Upper case: AmNA; Lower case: DNA; Linkage: all phosphorothioated

Table S2 Sequences of LNA-modified ASO

ID	No.	Sequences	ID	No.	Sequences	ID	No.	Sequences
HsPCSK9-11-LNA(14)	1	CCgcctggagcTGa	HsPCSK9-821-LNA(14)	31	CTggaccagctGGc	HsPCSK9-1381-LNA(14)	61	GAgtgtgctgaCCa
HsPCSK9-21-LNA(14)	2	GCcaccaggacCGc	HsPCSK9-871-LNA(14)	32	CGgctgtacccACc	HsPCSK9-1441-LNA(14)	62	CTcagcagctcCTc
HsPCSK9-61-LNA(14)	3	GGacccaggagCAg	HsPCSK9-961-LNA(14)	33	TAgaggcaggcATc	HsPCSK9-1451-LNA(14)	63	ACtggagcagcTCa
HsPCSK9-121-LNA(14)	4	AAggctagcacCAg	HsPCSK9-971-LNA(14)	34	GGctggggagtAGa	HsPCSK9-1461-LNA(14)	64	TCctggagaaaCTg
HsPCSK9-181-LNA(14)	5	TGgaaggtggcTGt	HsPCSK9-991-LNA(14)	35	GTgatgacctcGGg	HsPCSK9-1471-LNA(14)	65	CGcttcccactCCt
HsPCSK9-201-LNA(14)	6	ACggatccttgGCg	HsPCSK9-1001-LNA(14)	36	GGccccaactgTGa	HsPCSK9-1501-LNA(14)	66	CCcccttgggcCTc
HsPCSK9-241-LNA(14)	7	TCctccttcagCAc	HsPCSK9-1011-LNA(14)	37	GGgcattggtgGCc	HsPCSK9-1541-LNA(14)	67	CTcacccccaaAAg
HsPCSK9-311-LNA(14)	8	GAggtatccccGGc	HsPCSK9-1041-LNA(14)	38	CCaaagtccccAGg	HsPCSK9-1551-LNA(14)	68	CGtagacacccTCa
HsPCSK9-321-LNA(14)	9	GGatcttggtgAGg	HsPCSK9-1051-LNA(14)	39	AAgttggtcccCAa	HsPCSK9-1561-LNA(14)	69	CTggcaatggcGTa
HsPCSK9-331-LNA(14)	10	AAgacatgcagGAt	HsPCSK9-1071-LNA(14)	40	AGaggtccacaCAg	HsPCSK9-1571-LNA(14)	70	CAggcagcaccTGg
HsPCSK9-351-LNA(14)	11	AGccaggaagaAGg	HsPCSK9-1081-LNA(14)	41	CCtggggcaaaGAg	HsPCSK9-1581-LNA(14)	71	CCtggggtagcAGg
HsPCSK9-361-LNA(14)	12	TTcaccaggaaGCc	HsPCSK9-1091-LNA(14)	42	GAtgtcctcccCTg	HsPCSK9-1611-LNA(14)	72	CTggtggagctGTg
HsPCSK9-371-LNA(14)	13	GCcactcatctTCa	HsPCSK9-1101-LNA(14)	43	AGgcaccaatgATg	HsPCSK9-1631-LNA(14)	73	GGtccccatgcTGg
HsPCSK9-431-LNA(14)	14	AGaggagtcctCCt	HsPCSK9-1121-LNA(14)	44	GCaggtgctgcAGt	HsPCSK9-1671-LNA(14)	74	AGcctgtgaggACg
HsPCSK9-441-LNA(14)	15	GGgcaaagacaGAg	HsPCSK9-1131-LNA(14)	45	GTgacacaaagCAg	HsPCSK9-1681-LNA(14)	75	TGggagctgcaGCc
HsPCSK9-451-LNA(14)	16	GGgatgctctgGGc	HsPCSK9-1161-LNA(14)	46	CGtgggcagcaGCc	HsPCSK9-1691-LNA(14)	76	CAcctcccagtGGg
HsPCSK9-501-LNA(14)	17	GGtattcatccGCc	HsPCSK9-1171-LNA(14)	47	ATgccagccacGTg	HsPCSK9-1701-LNA(14)	77	CAaggtcctccACc
HsPCSK9-531-LNA(14)	18	CCtccaccaggCTg	HsPCSK9-1181-LNA(14)	48	CAtggctgcaaTGc	HsPCSK9-1711-LNA(14)	78	TTgtgggtgccAAg
HsPCSK9-541-LNA(14)	19	AGgagatacacCTc	HsPCSK9-1191-LNA(14)	49	CAgacagcatcATg	HsPCSK9-1731-LNA(14)	79	GTggcctcagcACa
HsPCSK9-551-LNA(14)	20	GCtggtgtctaGGa	HsPCSK9-1231-LNA(14)	50	AGtctctgcctCAa	HsPCSK9-1771-LNA(14)	80	CTggcctccctGTg
HsPCSK9-561-LNA(14)	21	CActctgtatgCTg	HsPCSK9-1241-LNA(14)	51	GAagtggatcaGTc	HsPCSK9-1791-LNA(14)	81	GGcagcaggaaGCg
HsPCSK9-591-LNA(14)	22	CCatgaccctgCCc	HsPCSK9-1251-LNA(14)	52	CTttggcagagAAg	HsPCSK9-1801-LNA(14)	82	CCtggggcatgGCa
HsPCSK9-641-LNA(14)	23	TCtgtggaagcGGg	HsPCSK9-1261-LNA(14)	53	TTgatgacatcTTt	HsPCSK9-1811-LNA(14)	83	GCattccagacCTg
HsPCSK9-651-LNA(14)	24	TGctggcctgtCTg	HsPCSK9-1271-LNA(14)	54	CCaggcctcatTGa	HsPCSK9-1821-LNA(14)	84	CCttgactttgCAt
HsPCSK9-661-LNA(14)	25	CTgtcacacttGCt	HsPCSK9-1281-LNA(14)	55	CCtcagggaacCAg	HsPCSK9-1831-LNA(14)	85	ATtccatgctcCTt
HsPCSK9-681-LNA(14)	26	CTgccaggtggGTg	HsPCSK9-1301-LNA(14)	56	GGgggtcagtaCCc	HsPCSK9-1851-LNA(14)	86	CCtgctcctgaGGg
HsPCSK9-691-LNA(14)	27	CTgaccaccccTGc	HsPCSK9-1311-LNA(14)	57	CCaccaggttgGGg	HsPCSK9-1911-LNA(14)	87	AGgtcccagggAGg
HsPCSK9-721-LNA(14)	28	GCacccttggcCAc	HsPCSK9-1331-LNA(14)	58	GGtgctgggggGCa	HsPCSK9-1931-LNA(14)	88	GTaggcccccaGGa
HsPCSK9-751-LNA(14)	29	CAgttgagcacGCg	HsPCSK9-1351-LNA(14)	59	AGctgccaaccTGc	HsPCSK9-1991-LNA(14)	89	GCtggtgctgcCTg
HsPCSK9-761-LNA(14)	30	CTtcccttggcAGt	HsPCSK9-1361-LNA(14)	60	CCtgcaaaacaGCt	HsPCSK9-2021-LNA(14)	90	GCagatggcaaCGg

Upper case: LNA; Lower case: DNA; Linkage: all phosphorothioated
